# Adult-Onset Hypokalemic Periodic Paralysis With the p.Arg672Cys Variant in the SCN4A Gene: A Case Report

**DOI:** 10.7759/cureus.113543

**Published:** 2026-07-28

**Authors:** Taro Okabe, Manabu Izumi, Isao Kouno, Seigo Yukisawa, Akari Matsuda

**Affiliations:** 1 Department of General Medicine, Saiseikai Utsunomiya Hospital, Utsunomiya, JPN; 2 Department of Medical Genetics Center, Saiseikai Utsunomiya Hospital, Utsunomiya, JPN

**Keywords:** adult-onset, case report, channelopathy, exercise-induced, flaccid paralysis, genetic testing, hypokalemic periodic paralysis, muscle weakness, scn4a gene

## Abstract

Hypokalemic periodic paralysis (HypoKPP) is a rare channelopathy that typically presents in adolescence with recurrent episodes of muscle weakness triggered by factors that lower serum potassium. We report a 37-year-old Japanese male with genetically confirmed HypoKPP caused by the rare SCN4A p.Arg672Cys variant. Despite late onset (early 30s) and infrequent attacks, the patient presented with classic flaccid paralysis and severe hypokalemia (1.8 mEq/L) triggered by exercise, alcohol consumption, and carbohydrate intake. Genetic testing confirmed the diagnosis after the condition was initially misattributed to insulin-related hypokalemia. This case highlights the importance of considering HypoKPP in adults with new-onset weakness and unexplained hypokalemia, even in atypical presentations characterized by late onset and infrequent attacks.

## Introduction

Hypokalemic periodic paralysis (HypoKPP) is a rare genetic disorder characterized by recurrent episodes of muscle weakness or paralysis associated with decreased serum potassium levels. With an estimated prevalence of one in 100000, it belongs to a group of disorders known as channelopathies, resulting from variants in genes encoding ion channels [1]. HypoKPP is predominantly caused by variants in the CACNA1S gene (encoding a calcium channel) or the SCN4A gene (encoding a sodium channel) and is inherited in an autosomal dominant manner [2].

Typical presentations include acute, reversible episodes of flaccid paralysis, often triggered by factors that lower serum potassium, such as high-carbohydrate meals, strenuous exercise followed by rest, emotional stress, or alcohol consumption [2,3]. Classical teaching suggests that symptoms usually begin in the first or second decade of life, with a peak incidence during adolescence [1].

However, clinical presentations can vary significantly, with some cases exhibiting atypical characteristics such as late onset, early onset, infrequent attacks, or unusual trigger patterns [2,4]. These variations may lead to diagnostic delays or misdiagnosis. Here, we report a case of genetically confirmed HypoKPP with the relatively rare SCN4A p.Arg672Cys variant, notable for its late onset (early 30s) and exceptionally infrequent attacks despite significant precipitating factors.

## Case presentation

A 37-year-old Japanese male was brought to our hospital by ambulance at 6:30 a.m. with flaccid weakness in his extremities from the time he woke up. The day before admission, he participated in a sporting event from 2:00 p.m. to approximately 3:00 p.m. He subsequently consumed a large dinner, including alcoholic beverages and sweet foods. Additionally, he ate a late-night meal at 1:00 a.m. on the day of admission. He had a history of four hospital visits four to five years before the current admission for post-exercise episodes of weakness associated with hypokalemia, which occurred after heavy meals or alcohol consumption and were previously attributed to insulin-related hypokalemia at another institution. Serum potassium levels were documented during two of these episodes and measured 1.7 mEq/L and 1.9 mEq/L, respectively. He had no symptoms during the interictal period and had not experienced a weakness attack during the previous four years. He rarely consumed alcohol, drinking approximately 700 mL of beer once every two weeks. He had no history of medication use, including diuretics, laxatives, or other drugs that could cause hypokalemia, and no gastrointestinal symptoms such as vomiting or diarrhea.

**Figure 1:**
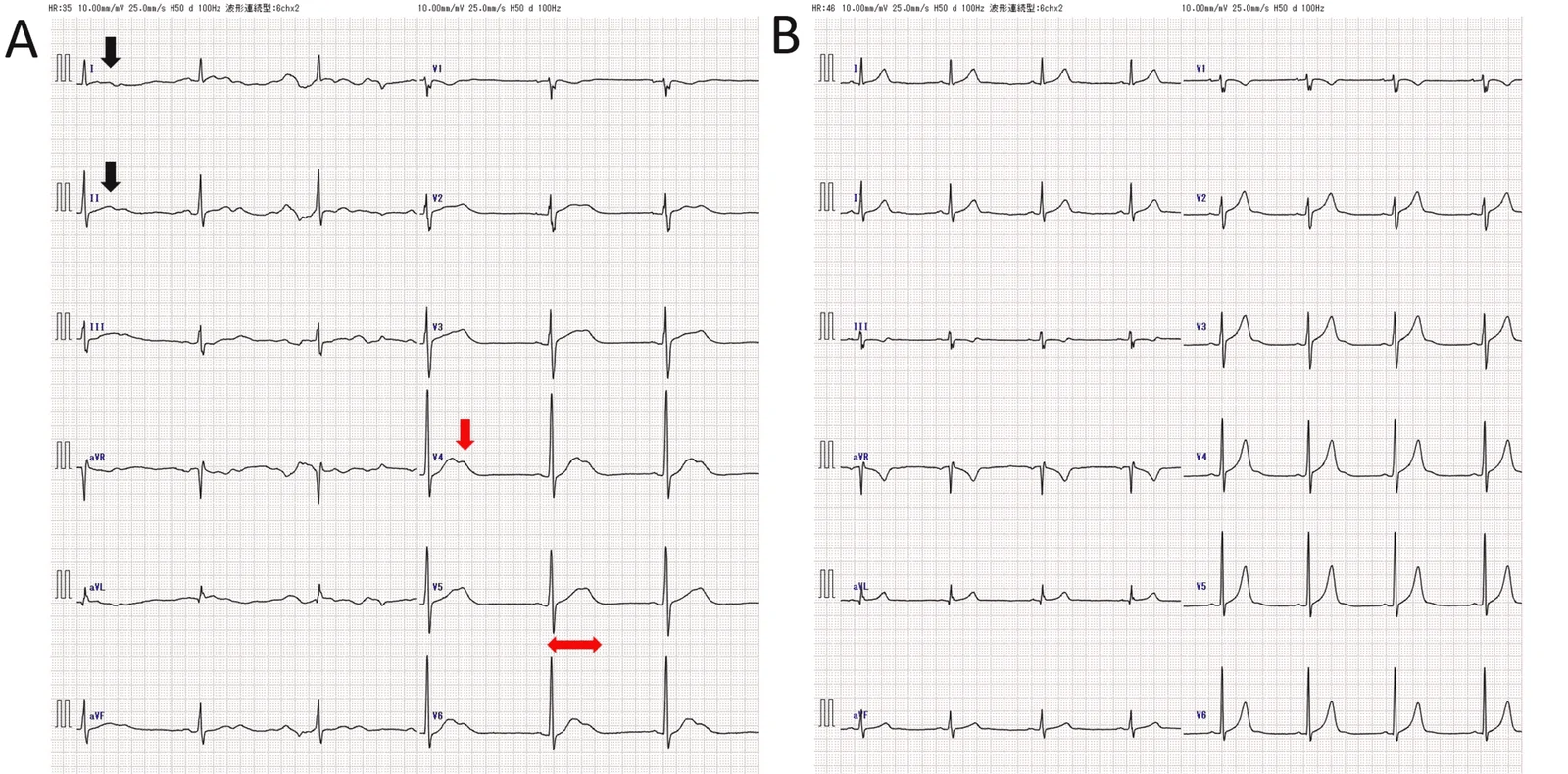
Electrocardiogram at presentation The electrocardiogram at presentation (A) demonstrated sinus bradycardia with a heart rate of 35 beats/min, along with T-wave flattening (black arrow), prominent U waves (red arrow), and a prolonged corrected QT interval (QTc), calculated using Bazett's formula (QTc=QT/√RR), of 537 msec (red double-headed arrow). After potassium correction (serum potassium level, 4.2 mEq/L), the electrocardiogram returned to normal (B).

**Figure 2:**
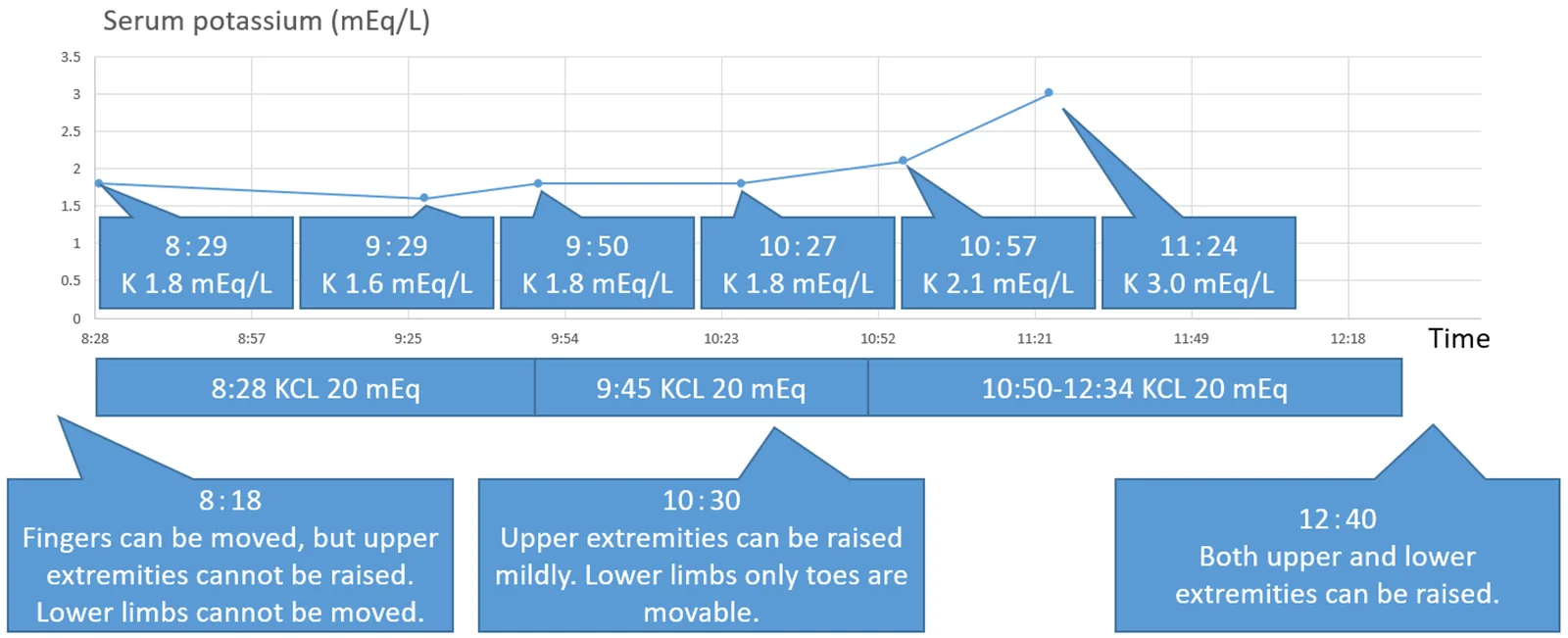
Time course of serum potassium levels and clinical improvement during potassium replacement Notably, clinical improvement appeared to precede the rise in serum potassium. After initiation of KCl infusion (8:28; K, 1.8 mEq/L), the serum potassium level did not increase and, if anything, decreased slightly, remaining in the 1.6-1.8 mEq/L range through 9:50. Despite this persistently low potassium level, muscle strength was already recovering steadily: whereas only the fingers could be moved at 8:18, by 10:30 (K, still 1.8 mEq/L and well within the hypokalemic range), the patient could mildly raise the upper extremities and move the toes of the lower extremities. In other words, meaningful clinical recovery had already begun while the serum potassium level remained dangerously low. A definite rise in serum potassium was not observed until 10:57 (2.1 mEq/L), more than two hours after clinical improvement was first observed, reaching 3.0 mEq/L by 11:24, with recovery of both upper and lower extremity elevation by 12:40. K: serum potassium; KCl: potassium chloride

On admission, the patient was alert and appeared well, with no difficulty in breathing. His body temperature was 36.2°C, blood pressure was 116/74 mmHg, heart rate was 35 beats/min, and respiratory rate was 18 breaths/min. Physical examination findings were unremarkable except for flaccid paralysis of the extremities. Laboratory findings on admission were as follows: white blood cell count, 7070/μL; hemoglobin, 14.7 g/dL; platelet count, 218000/μL; blood urea nitrogen, 13.7 mg/dL; creatinine, 0.84 mg/dL; serum sodium, 144 mEq/L; serum potassium, 1.8 mEq/L; and serum magnesium, 1.9 mEq/L. His free thyroxine level was 1.18 ng/dL, thyroid-stimulating hormone level was 3.2 µIU/mL, creatine phosphokinase level was 4911 IU/L, and glucose level was 99 mg/dL (Table 1). Electrocardiography showed sinus bradycardia with a heart rate of 35 beats/min and a prolonged corrected QT interval of 537 msec (Figure 1), which normalized after potassium correction. Chest radiography and abdominal ultrasonography findings were unremarkable. After approximately 60 mEq of potassium was administered intravenously over three hours, the serum potassium level increased to 3.0 mEq/L, with concomitant improvement in limb weakness (Figure 2). The creatine phosphokinase level, which was 4911 IU/L on admission, subsequently decreased to 3063 IU/L on the day after admission, 1159 IU/L two days after admission, and 147 IU/L 10 days after admission. The patient remained hospitalized for observation, during which there was no decrease in the serum potassium level or recurrence of limb weakness. Serum potassium levels over the following days remained between 4.0 and 4.5 mEq/L in the absence of additional treatment.

**Table 1:** Initial laboratory data of the patient HCO₃^-^: bicarbonate

Blood and urinary studies	Patient values	Reference values
White blood cell count	7070	3300-8600 /μL
Hemoglobin level	14.7	13.7-16.8 g/dL
Platelet count	218000	158000-348000 /μL
Blood urea nitrogen	13.7	8.0-20.0 mg/dL
Serum creatinine	0.84	0.65-1.07 mg/dL
Serum sodium	144	138-145 mEq/L
Serum potassium	1.8	3.6-4.8 mEq/L
Serum chloride	109	98-106 mEq/L
Serum magnesium	1.9	1.8-2.4 mEq/L
Free thyroxine level	1.18	0.90-1.70 ng/dL
Thyroid stimulating hormone level	3.2	0.5-5.0 µIU/mL
Creatine phosphokinase level	4911	59-248 IU/L
Glucose level	99	66-93 mg/dL
pH	7.362	7.32-7.42
HCO_3_^-^	25.1	24.0-28.0 mmol/L
Base excess	-0.4	-2-2 mEq/L
Urinary potassium	7.0	15-40 mEq/L
Urinary creatinine	87.6	20-300 mg/dL

We suspected HypoKPP based on the patient's clinical history and performed genetic testing, which revealed a heterozygous SCN4A p.Arg672Cys variant, along with a second heterozygous SCN4A variant (p.Arg18Cys) with conflicting classifications of pathogenicity (Table 2). Needle electromyography performed after discharge showed no myotonic discharges, and the prolonged exercise test showed no obvious attenuation.

**Table 2:** Genetic variant summary, SCN4A (NM_000334.4) Methods: Targeted NGS (hybridization capture) covering the coding exons and flanking intronic regions (≤10 bp) of CACNA1S, SCN4A, KCNJ2, and KCNJ5, aligned to GRCh38/hg38. Variants absent from population databases or with an allele frequency ≤0.1% were reported. Note: Classifications above are from ClinVar as cited in the report, not an independently performed ACMG-AMP assessment. NGS: next-generation sequencing; ACMG: American College of Medical Genetics and Genomics; AMP: Association for Molecular Pathology

Variant	HGVS.c	HGVS.p	Zygosity	ClinVar classification
1	c.2014C>T	p.Arg672Cys	Heterozygous	Pathogenic
2	c.52C>T	p.Arg18Cys	Heterozygous	Conflicting classifications of pathogenicity

He was discharged from the hospital with a prescription for potassium chloride to be taken in the event of another episode of muscle weakness and was advised to avoid excessive exercise and overeating. Following this advice, his serum potassium levels remained stable at 4.6 mEq/L one week after discharge, 4.5 mEq/L one month after discharge, and 4.5 mEq/L four months after discharge. Genetic counseling was provided regarding the characteristics of HypoKPP, lifestyle precautions, and its autosomal dominant inheritance pattern. The patient has no children and no known family history of similar symptoms. No recurrence of symptoms has been observed during follow-up. This patient's clinical presentation combined several classic diagnostic features of HypoKPP with a number of atypical, case-specific characteristics, as summarized in Table 3.

**Table 3:** Clinical phenotype summary K: serum potassium

Classic diagnostic features present in this case:
Acute flaccid limb paralysis with severe hypokalemia (serum K⁺ 1.8 mEq/L)
Attacks triggered by strenuous exercise, a heavy/high-carbohydrate meal, and alcohol
Rapid, near-complete resolution of weakness with potassium repletion
Asymptomatic interictal periods with normalization of serum potassium between attacks
Autosomal dominant channelopathy confirmed by genetic testing (SCN4A p.Arg672Cys)
Unremarkable interictal electrodiagnostic studies (no myotonic discharge on EMG; no attenuation on prolonged exercise test)
Atypical features specific to this patient:
Late onset of first attack (age 32) rather than the typical first or second decade
Markedly infrequent attacks, only five episodes over roughly five years, despite recurrent exposure to known triggers
Apparent clinical improvement in weakness preceding the measured rise in serum potassium (see Discussion for a cautious interpretation)

## Discussion

HypoKPP is a disorder in which patients experience paralytic attacks associated with hypokalemia. Serum potassium levels are typically less than 3.5 mEq/L, and paralytic attacks are characterized by decreased muscle tone that is more pronounced proximally than distally [2]. Known triggering factors include strenuous exercise and carbohydrate-rich nighttime meals, both of which were present in this case.

Interestingly, the patient's weakness appeared to begin improving before a substantial rise in serum potassium was documented, although serum potassium was not measured continuously, and this apparent sequence should be interpreted with caution. One possible contributing factor is that only a small fraction of total body potassium (roughly 10%) resides in the extracellular compartment, so relatively modest shifts in potassium distribution between the intracellular and extracellular spaces could plausibly produce clinically meaningful changes in neuromuscular function without being fully captured by intermittent serum sampling [5]. The appropriately low urinary potassium excretion (7.0 mEq/L) in the setting of severe hypokalemia argues against renal potassium wasting and supports a transcellular shift as the primary mechanism, consistent with the diagnosis of periodic paralysis.

Although the disease is hereditary, the age at onset ranges from five to 35 years [1]. In a systematic review by Latorre et al. encompassing 40 cases of HypoKPP, the mean age at onset was 15.3±9.7 years [6]. The interval between attacks is also variable, and it is important to obtain a medical history extending back several years [2].

Distinguishing HypoKPP from thyrotoxic periodic paralysis, an important acquired mimic of adult-onset hypokalemic paralysis, is essential in this age group, because thyrotoxic periodic paralysis resolves once thyroid function is corrected and does not require genetic testing. In this patient, normal thyroid function test results argued against this diagnosis.

Needle electromyography performed after discharge showed no myotonic discharges, and the prolonged exercise test showed no obvious attenuation of the compound muscle action potential. These essentially normal interictal findings should not be interpreted as evidence against the diagnosis: both studies are frequently unremarkable between attacks, even in genetically confirmed sodium channel periodic paralysis, and the identification of the SCN4A p.Arg672Cys variant provides a definitive molecular diagnosis independent of these electrophysiological findings.

The markedly elevated creatine phosphokinase level on admission (4,911 IU/L) most likely reflects skeletal muscle membrane injury related to the preceding strenuous exercise and the paralytic episode itself, a pattern also described in other reports of periodic paralysis. Severe hypokalemia may also cause capillary constriction, reducing blood supply to the muscles and thereby contributing to muscle cell damage [7]. Notably, Young-Lee Jung et al. reported a case of HypoKPP in which the creatine phosphokinase level increased markedly to 45720 IU/L, illustrating that substantially greater degrees of muscle injury can occur in this condition [7].

The SCN4A gene accounts for only 8.6% to 9.6% of HypoKPP cases, making it a comparatively rare genetic cause of this already rare disease [8]. A second heterozygous SCN4A variant, p.Arg18Cys, was also identified; however, this variant has conflicting classifications of pathogenicity in ClinVar, and the patient's phenotype is therefore attributed primarily to the well-established pathogenic p.Arg672Cys variant. The p.Arg672Cys variant was first reported in a Korean family with incomplete penetrance among female carriers [9]. This variant is particularly rare; a Chinese study reported only one case among 71 patients with sporadic HypoKPP [10]. In Japan, a recent study of 119 patients with suspected periodic paralysis achieved a molecular diagnostic rate of only 26.2%, and none of the genetically confirmed cases harbored the p.Arg672Cys variant [11]. Therefore, there are few reports summarizing the clinical course of patients with this specific variant.

To our knowledge, no case involving the p.Arg672Cys variant has previously been reported in the Japanese-language medical literature, underscoring the regional novelty of this report. This case represents a valuable example of adult-onset HypoKPP associated with the p.Arg672Cys variant in the Japanese population.

## Conclusions

In this case, four previous episodes of weakness and hypokalemia occurring four to five years earlier were attributed to insulin-induced hypokalemia, and a hereditary channelopathy was not considered at that time. Given that HypoKPP can present between the ages of five and 35 years, it may be overlooked in the differential diagnosis of adult-onset weakness, as occurred in this patient, who first developed symptoms at age 32 and was not genetically diagnosed until a subsequent admission.

Rather than being considered broadly in all cases of adult-onset weakness without electrolyte abnormalities, HypoKPP should specifically be included in the differential diagnosis of adults presenting with recurrent, otherwise unexplained episodes of acute hypokalemic flaccid paralysis, particularly when attacks are triggered by strenuous exercise or carbohydrate-rich meals, even in the absence of a known family history.
